# The Role of Cashew (*Anacardium occidentale* L.) Nuts on an Experimental Model of Painful Degenerative Joint Disease

**DOI:** 10.3390/antiox9060511

**Published:** 2020-06-10

**Authors:** Roberta Fusco, Rosalba Siracusa, Alesso Filippo Peritore, Enrico Gugliandolo, Tiziana Genovese, Ramona D’Amico, Marika Cordaro, Rosalia Crupi, Giuseppina Mandalari, Daniela Impellizzeri, Salvatore Cuzzocrea, Rosanna Di Paola

**Affiliations:** 1 Department of Chemical, Biological, Pharmaceutical and Environmental Sciences, University of Messina, 98166 Messina, Italy; rfusco@unime.it (R.F.); rsiracusa@unime.it (R.S.); aperitore@unime.it (A.F.P.); egugliandolo@unime.it (E.G.); tgenovese@unime.it (T.G.); rdamico@unime.it (R.D.); gmandalari@unime.it (G.M.); dipaolar@unime.it (R.D.P.); 2 Department of Biomedical, Dental and Morphological and Functional Imaging University of Messina, Via Consolare Valeria, 98125 Messina, Italy; cordarom@unime.it; 3 Department of Veterinary Sciences, University of Messina, 98168 Messina, Italy; rcrupi@unime.it; 4 Department of Pharmacological and Physiological Science, Saint Louis University School of Medicine, Saint Louis, MO 63104, USA

**Keywords:** osteoarthritis, cashew nuts, antioxidant

## Abstract

Osteoarthritis is a progressive joint disease characterized by the activation of different molecular mediators, including proinflammatory cytokines, reactive oxygen species, metalloproteinases and nociceptive mediators. *Anacardium occidentale* L. is a medicinal plant with anti-oxidative and anti-inflammatory properties. In this study we evaluate the effects of cashew nuts (from *Anacardium occidentale* L.) oral administration on an experimental model of painful degenerative joint disease. Monosodium iodoacetate (MIA) was intraarticularly injected, and cashew nuts were orally administered three times per week for 21 days, starting the third day after MIA injection. Nociception was evaluated by a Von Frey filament test, and motor function by walking track analysis at 3, 7, 14 and 21 days after osteoarthritis. Histological and biochemical alteration were examined at the end of the experiment. Cashew nuts administration reduced pain-like behavior and showed antioxidant activities, restoring biochemical serum parameters: glutathione (GSH), catalase (CAT) levels, glutathione peroxidase (GPx) activity and lipid peroxidation. Moreover, cashew nuts ameliorated radiographic and histological alteration, resulting in decreased cartilage degradation, pro-inflammatory cytokines and metalloproteinases levels and mast cells recruitment. Our results demonstrated that the oral assumption of cashew nuts counteracts the inflammatory and oxidative process involved in osteoarthritis.

## 1. Introduction

Osteoarthritis is one of the most leading causes of disability. Worldwide evaluations show that 18% of women and 9.6% of men ≥60 years are affected by osteoarthritis [1]. It is a complex pathology characterized by an inflammatory mediators storm released by bone, cartilage and synovium [2].

These mediators activate nociceptive and degradative pathways associated with the development of the disease [2,3]. Allodynia is a prominent symptom of osteoarthritis, which represents a debilitating characteristic and is often treatment-resistant. Thus, pathology modification strategies for reversing or arresting the development of joint degeneration have been suggested to impact on pain generation. Emerging views consider osteoarthritis no longer as predominantly a cartilage issue, but as a complex disease characterized by a progressive joint destruction. These processes are induced by pro-inflammatory mediators responsible for releasing proteolytic enzymes and degrading the extracellular matrix. Redox mechanisms influence intracellular signaling osteoarthritis progression [4,5]. The concentrations of several reactive oxygen species (ROS), oxidized and reduced thiols, the oxidative stress indicator and the correlated enzymes are monitored in osteoarthritis. Altindag and colleagues showed lower serum catalase activity and thiol levels in osteoarthritis patients, compared to controls [6]. Moreover, these patients show a higher oxidative stress index, which negatively correlated with prolidase activity levels. Regan and colleagues reported lower levels of extracellular superoxide dismutase (SOD), ascorbate and glutathione in osteoarthritis patients [7]. A diet rich of flavonoids, polyphenols, vegetables and fresh fruit counteracts the oxidative effect of ROS and have important effects on osteoarthritis [8,9]. Recent studies proposed antioxidants with further anti-inflammatory action as therapeutic tools in osteoarthritis [1]. Preclinical and clinical studies describe antioxidant and antimicrobial properties of medicinal plants [10]. *Anacardium occidentale* L. is a well-studied medicinal plant with a therapeutic effect. Different ethnopharmacological applications have been described for the various portions of the plant (flowers, stem, fruits and leaves), [11]. In particular, its fruits (cashew nuts) have important antioxidant activity [12]. In a xanthine/hypoxanthine oxidase test, cashew nuts show elevated antioxidant capacity with 94% inhibition from the fiber and 100% inhibition from nut liquid extract of the nut. Lipid peroxidation levels show a 95% rise in total antioxidant capacity and 80% reduction in the formation of malondialdehyde [13]. Moreover, cashews are able to reduce lipid peroxidation and raise SOD and catalase (CAT) activity, reducing injuries to cell membranes [14]. The monosodium iodoacetate (MIA) animal model is an established model of osteoarthritis for both pain-related behaviors and histopathological changes [15,16,17,18]. Accordingly, our study was designed to evaluate the potential beneficial effect of the oral administration of cashew nuts on an experimental rat model of MIA-induced osteoarthritis.

## 2. Materials and Methods

### 2.1. Animals

Male rats (Sprague–Dawley (200–230 g, Envigo, Milan, Italy)) were used throughout. The University of Messina Review Board for animal care (OPBA) approved the study (500/2018-PR). All animal experiments agree with the new Italian regulations (D.Lgs 2014/26), EU regulations (EU Directive 2010/63) and the ARRIVE guidelines.

### 2.2. Experimental Protocol

Osteoarthritis was induced by MIA injection in the knee joint [18]. On day 0, rats were anesthetized and a volume of 25 µL sterile saline solution + 3 mg of MIA was injected into the knee joint. Rats were administered with vehicle (saline) or cashew nuts (100 mg/kg) as a repeated administration three times per week for 21 days, starting the third day after MIA injection. On day 21 post-MIA administration, blood samples were collected and knee joints were inspected in detail to determine histopathological changes. Cartilage was stained to verify whether there was any presence of osteoarthritis [17,19].

### 2.3. Experimental Groups

Rats were randomly divided into the following groups:

(1). MIA + vehicle (saline): rats subjected to surgery as described above, and administered with vehicle (*n* = 10)
(2). MIA + cashew nuts (100 mg/kg): rats subjected to surgery as described above, and administered with cashew nuts (100 mg/kg) (*n* = 10)
(3). SHAM groups. rats were administered by intra-articular injection with 0.9% saline instead of’ MIA, and were treated with either vehicle or cashew nuts.

The tested dose was chosen based on previous studies performed in our laboratories [12]. Rats were sacrificed at twenty-one days, and after surgical procedures, blood was collected and knee joints were harvested for histological investigation.

### 2.4. Serum Enzyme Measurements

At the end of the experiment, blood samples were collected, allowed to coagulate at room temperature for 30 min and separated by centrifugation at 3000 r.p.m. for 15 min. The clean, clear serum was separated [20]. Reduced Glutathione (GSH), glutathione peroxidase (GPx), l-malondialdehde (MDA) [21], catalase (CAT) and super oxide dismutase (SOD) were assessed according to the method described [22,23,24,25].

### 2.5. Measurement of Cytokines, Metalloproteinases and NGF

The levels of interleukin-1beta (IL-1β), tumor necrosis factor alpha (TNF-α), nerve growth factor (NGF), and matrix metalloproteinase-1-3-9 (MMP-1 MMP-3 MMP-9) were investigated in serum. Assays were performed using commercial colorimetric ELISA kits (IL-1β, TNF-α, NGF: Thermo Fisher Scientic, Monza, Italy; MMP-3, MMP-1, MMP-9: Cusabio, Houston, TX, USA ).

### 2.6. Pain Measurement

Mechanical allodynia was assessed using an electronic Von Frey test (BIO-EVF4, Bioseb, Vitrolles, France), as previously described [19,26]. The weight needed to have a paw withdrawal reflex (grams) and the time between the stimulus and the response (seconds) were automatically identified and recorded. A maximum weight of 50 g and a ramp speed of 20 s were used as cut off for the test.

### 2.7. Analysis of Motor Function (Walking Track Analysis)

Motor functional recovery was investigated by walking track analysis, as previously described [19]. Walking track analysis was evaluated before MIA injection, and 3, 7, 14 and 21 days after. The measurements recorded were the following: (i) the print length (PL, i.e., the distance from the heel to toe), (ii) the toe spread (TS, i.e., the distance from the first to the fifth toes), (iii) the intermediary toe spread (IT, i.e., the distance from the second to fourth toes). These measures were noted for the MIA-injected and contralateral limb, with the prefix E and N being added, respectively. The motor functional recovery was calculated using the following formula, whose numerical value is termed SFI (sciatic functional index): −38.3 [(EPL − NPL)/NPL] + 109.5 [(ETS − NTS)/NTS] + 13.3 [(EIT − NIT)/NIT] − 8.8 [27]. SFI values in the control group were assumed as zero.

### 2.8. Radiographic Analysis

Radiographic analysis was performed by X-ray (Bruker FX Pro instrument, Milan, Italy). A semiquantitative grading scale was employed to evaluate the radiographs [28]. Briefly, the study features were scored as follows: joint space, from 0 (normal) to 3 (complete loss of joint space); subchondral bone sclerosis, from 0 (normal) to 3 (severe); osteophyte formation, from 0 (normal) to 3 (osteophytes present on both tibial and femoral condyle).

### 2.9. Histological Analysis

Tibiofemoral joints were harvested twenty-one days after MIA injection and fixed in neutral buffered formalin, as previously described [18]. Tissue sections were stained with hematoxylin and eosin (H&E), observed using a Leica DM6 microscope at ×10 magnification (Leica Microsystems SpA, Milan, Italy) associated with Leica LAS X Navigator software (Leica Microsystems SpA, Milan, Italy). Collagen content was evaluated by Masson’s trichrome according to the manufacturer’s instruction (Bio-Optica, Italy, Milan). Toluidine blue staining was used to evaluate the mast cells’ number and cartilage degeneration [18,29]. Modified Mankin histologic scoring system was employed to evaluate cartilage damage [30].

### 2.10. Reagents

Cashew kernel samples (*Anacardium occidentale* L.) obtained from West Africa were used in the study. Their proximal composition has been previously reported [12]. All other materials were purchased from Sigma-Aldrich Co. Stock solutions were prepared in nonpyrogenic saline (0.9% NaCl, Baxter Healthcare Ltd., Thetford, Norfolk, UK).

### 2.11. Data Analysis

All values in the figures and text are expressed as mean ± standard error of the mean (SEM) of *N* = 10 number of animals. In those experiments involving histology, the exhibited pictures are representative of at least three experiments performed on different days. Results were analyzed by one-way or two-way ANOVA, followed by a Bonferroni post-hoc test for multiple comparisons. A *p*-value < 0.05 was considered significant. * *p* < 0.05 vs. sham-vehicle, # *p* < 0.05 vs. MIA-vehicle, ** *p* < 0.01 vs. sham-vehicle, ## *p* < 0.01 vs. MIA-vehicle, *** *p* < 0.001 vs. sham-vehicle, ### *p* < 0.001 vs. MIA-vehicle.

## 3. Results

### 3.1. Effect of Cashew Nuts on Motor Function Deficits and Pain Induced by MIA Injection

**Figure 1:**
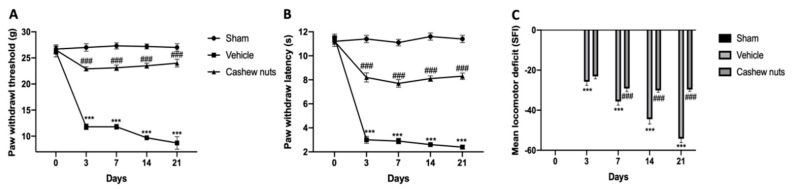
Effect of cashew nuts on monosodium iodoacetate (MIA)-induced pain-like behavior: Von Frey hair assessment test, the paw withdrawal threshold (PWT) (**A**), paw withdrawal latency (PWL) (**B**); walking track analysis (**C**). A *p*-value < 0.05 was considered significant. *** *p* < 0.001 vs. sham-vehicle, ### *p* < 0.001 vs. MIA-vehicle.

Because pain is the main sign of osteoarthritis, the secondary tactile allodynia was evaluated in MIA-injected animals. At days 3, 7, 14 and 21, the von Frey hair test showed reduced PWL and PWT in vehicle-treated rats compared to the sham (Figure 1A,B). Cashew nuts’ oral administration significantly reduced osteoarthritis allodynia, showing an antinociceptive property. Moreover, walking track analysis was performed to evaluate motor function at different time points. The control group was assumed as zero (Figure 1C). In the vehicle-treated animals, their locomotor function was significantly impaired, as shown by the SFI values lower than zero. Treatment with cashew nuts significantly improved joint mobility, thus reducing physical disability.

### 3.2. Effect of Cashew Nuts on Oxidative Stress Induced by MIA Injection

**Figure 2:**
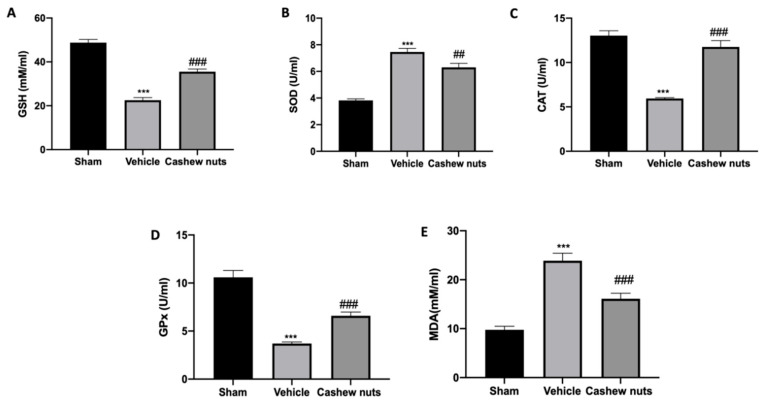
Effect of cashew nuts on antioxidant systems: glutathione (GSH) (**A**), super oxide dismutase (SOD) (**B**) and catalase (CAT) (**C**) serum levels; glutathione peroxidase (GPx) activity (**D**) and l-malondialdehde (MDA) level (**E**). A *p*-value < 0.05 was considered significant. ## *p* < 0.01 vs. MIA-vehicle, *** *p* < 0.001 vs. sham-vehicle, ### *p* < 0.001 vs. MIA-vehicle.

In order to evaluate the antioxidant effect of cashew nuts’ oral administration on osteoarthritis biochemical blood parameters were evaluated. GSH and CAT levels and GPx activity were reduced in the serum of MIA vehicle-treated animals as compared to the sham group. Treatment with cashew nuts normalized GSH and CAT levels and GPx activity (Figure 2A,C,D respectively). SOD levels were substantially increased by MIA injection and reduced by cashew nuts’ treatment (Figure 2B). Moreover, a significant increase in MDA level was detected in MIA vehicle-treated animals when compared to the control group. Cashew nuts’ administration decreased the enhanced MDA levels induced by MIA (Figure 2E).

### 3.3. Effects of Cashew Nuts on Inflammatory, Matrix Degradation and Nociceptive Markers Induced by MIA Injection

**Figure 3:**
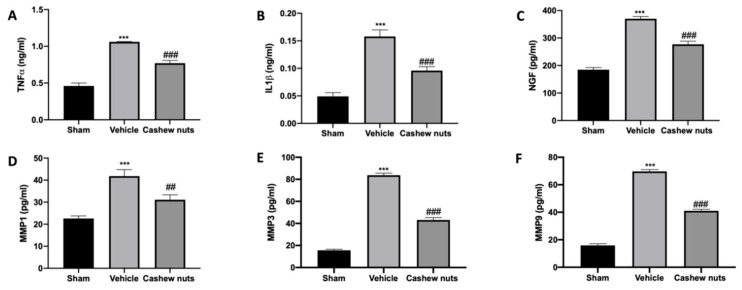
Effect of cashew nuts’ proinflammatory cytokines, metalloproteinases and mast cells infiltration: TNF-α (**A**), IL-1β (**B**), NGF (**C**), MMP-1 (**D**), MMP-3 (**E**), MMP-9 (**F**) serum levels. A *p*-value < 0.05 was considered significant. *** *p* < 0.001 vs. sham-vehicle, ## *p* < 0.01 vs. MIA-vehicle, ### *p* < 0.001 vs. MIA-vehicle.

To test whether cashew nuts’ administration may modulate pro-inflammatory cytokines’ expressions, levels of TNF-α (Figure 3A), IL-1β (Figure 3B) and NGF (Figure 3C), were measured. MIA Vehicle-treated animals showed increased inflammatory and nociceptive markers compared to the sham group. In contrast, cashew nuts’ treatment reduced these increases in serum levels. Moreover, we evaluated the expressions of matrix-degrading enzymes that play a key role in the destruction of articular cartilage induced by MIA injection. Serum levels of MMP-1 (Figure 3D), MMP-3 (Figure 3E) and MMP-9 (Figure 3F) were significantly increased in MIA vehicle-treated animals compared to the sham group. Cashew nuts’ administration reduced MMP-1, MMP-3 and MMP-9 expressions, respectively.

### 3.4. Effect of Cashew Nuts on Radiographic Joint Damage Induced by MIA Injection

**Figure 4:**
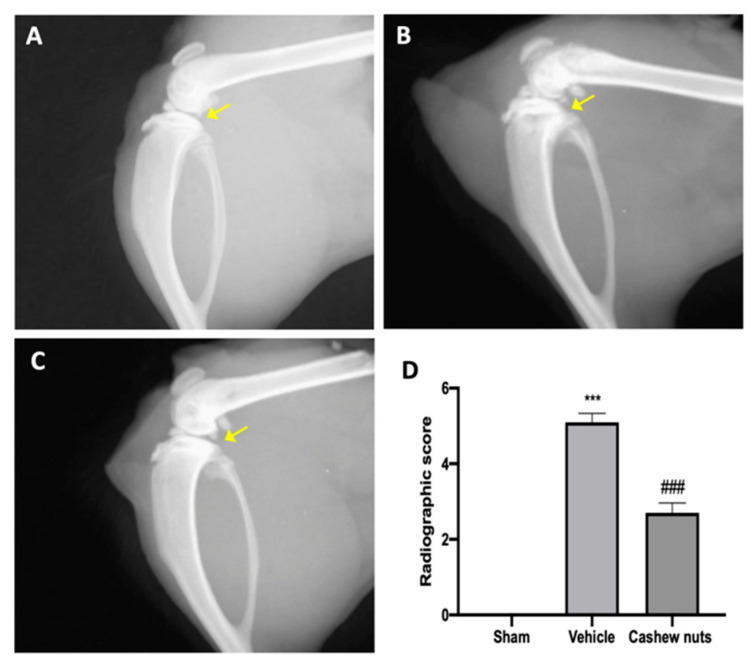
Effect of cashew nuts on radiographic analysis: sham (**A**), vehicle (**B**), cashew nuts (**C**), radiographic score (**D**). A *p*-value < 0.05 was considered significant. *** *p* < 0.001 vs. sham-vehicle, ### *p* < 0.001 vs. MIA-vehicle.

Radiographic analysis displayed joint damage around both the femur and tibia in MIA vehicle-treated animals (Figure 4B,D), with loss of joint space, articular cartilage erosion and subchondral bone sclerosis, as compared to sham animals (Figure 4A,D). Radiographies of cashew-nut-administered animals showed reduced collagen degradation and cartilage abnormalities (Figure 4C,D).

### 3.5. Effect of Cashew Nuts on Radiographic and Histologic Joint Damage Induced by MIA Injection

**Figure 5:**
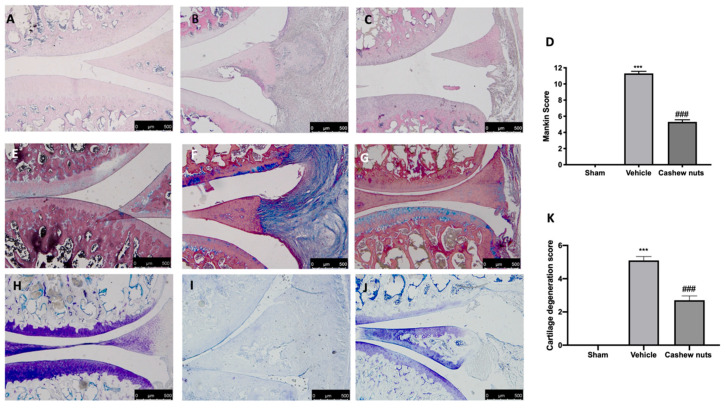
Effect of cashew nuts on histological changes and cartilage degradation: H&E staining: sham (**A**), vehicle (**B**), cashew nuts (**C**), Mankin score (**D**); Masson’s trichrome staining: sham (**E**), vehicle (**F**), cashew nuts (**G**); Toluidine blue staining: sham (**H**), vehicle (**I**), cashew nuts (J), cartilage degeneration score (**K**). A *p*-value < 0.05 was considered significant. *** *p* < 0.001 vs. sham-vehicle, ### *p* < 0.001 vs. MIA-vehicle.

Hematoxylin/eosin and Masson’s trichrome staining of knee sections from vehicle-treated animals 21 days after MIA injection showed multi-layering in transition, fibrillation and irregularities in the surface layer (Figure 5B,F), as compared to the control group (Figure 5A,E). Cashew nut administration resulted in a preservation of the joint architecture, reducing pannus formation and Mankin scores, accordingly (Figure 5C,D,G). Toluidine blue staining showed loss of proteoglycan and cartilage degradation in vehicle-treated animals (Figure 5I,K). In contrast, cashew nut administration protected cartilage integrity (Figure 5J,K).

### 3.6. Effects of Cashew Nuts on Mast Cell Infiltration Induced by MIA Injection

**Figure 6:**
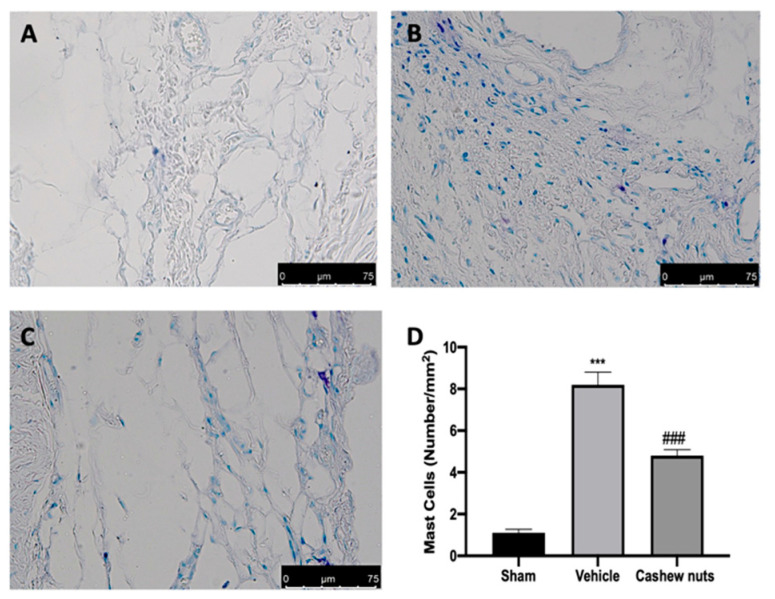
Effect of cashew nuts on mast cells infiltration: sham (**A**), vehicle (**B**), cashew nuts (**C**), mast cells count (**D**). A *p*-value < 0.05 was considered significant. *** *p* < 0.001 vs. sham-vehicle, ### *p* < 0.001 vs. MIA-vehicle.

Twenty-one days after MIA injection, vehicle-treated animals (Figure 6B,D) showed an increased number of infiltrated mast cells in the knee joint tissues, compared to the control group (Figure 6A,D). Cashew nuts’ administration reduced mast cell infiltration (Figure 6C,D).

## 4. Discussion

Often cited as evidence of joint inflammation in osteoarthritis is lipid mediators, cytokines and reactive oxygen species (ROS) release by chondrocytes, osteoblasts and synoviocytes, resulting in complex mechanical and biochemical interplay with other biological molecules to induce joint degeneration and promote pain [2,3]. The subchondral bone alterations results in pain severity and cartilage damage [31]. Chronic pain related with osteoarthritis is a major concern for which there are few viable treatments. In addition to pain and disability, osteoarthritis is associated with depression, comorbid conditions such as diabetes and an increased caregiver burden [32]. A variety of medications like nonsteroidal anti-inflammatories and opioids can cause severe side effects with limited benefits [33]. Total knee arthroplasty, although a definitive management, comes with a risk, such as postoperative infections, revisions and chronic pain. Newer injectable therapies are gaining attention as alternatives to medications because of a safer side effect profile, and are much less invasive than a joint replacement. Platelet-rich plasma is beginning to replace the more common injectable therapies of intra-articular corticosteroids and hyaluronic acid. New evidences suggest pain relief and functional improvement were obtained after the intra-articular hyaluronic acid, peptide and platelet-rich plasma injections in osteoarthritis, and decrease in pain was better in the peptide group [34]. Small studies have examined prolotherapy and stem cell therapy, and demonstrate some benefits.

Recently, the use of cryoanalgesia to create a cooled area around the nerves that innervate the knee has shown promise in treating knee-osteoarthritis [35]. Alternately, peripheral neuromodulation devices that use electricity to stimulate nerves and decrease sensation in painful joints could theoretically be used to treat knee-osteoarthritis and other nonoperative painful joints [36]. The use of multiomics, primarily proteomics, as well as metabolomics, is a new approach in osteoarthritis.

Previously, osteoarthritis was classified as a noninflammatory disease, but overwhelming evidences underline that the pro-inflammatory mediators released into the joint result in synovitis, producing nociceptive pain and peripheral sensitization [37,38,39,40]. Epidemiological data confirm that approximately 30% of patients with osteoarthritis suffer from neuropathic pain [41,42]. Thus, a therapeutic which can block neuropathy, inflammation and pain, is sorely needed. Oral supplementation with cashew nuts in MIA-injected rats resulted in an anti-allodynic effect and restored locomotor functionality. Free radicals amplify and mediate joint degeneration. The osteoarthritis progression is significantly linked to ROS and oxidative stress [43,44]. Several works demonstrate the decrease in GSH level [7], GPx activity and CAT levels in both patients [45,46] and osteoarthritic rats [20,47], confirming the role of oxidative stress in osteoarthritis pathogenesis. 

In parallel, the increase in SOD and MDA levels in the serum of osteoarthritic rats has been described [48]. Therapeutic strategies which are able to target the increased ROS represent effective intervention for osteoarthritis driven by oxidative stress [49]. Cashew nuts’ oral administration was able to restore GSH and CAT levels and GPx activity in MIA-injected animals. Moreover, these animals displayed reduced SOD and MDA levels. As mentioned before, several studies described the catabolic role of cytokines in osteoarthritis. In particular, TNF-α and IL-1β signaling, stimulating the activator protein 1 and nuclear factor-κB transcription factors, promotes the expression of several chrondrolytic and inflammatory mediators, such as metalloproteases (MMPs), and in turn, their autocrine production as well [37]. It has also been demonstrated that IL-1β overexpression increases NGF levels [38]. NGF, detected in osteoarthritis synovial fluid, is an important mediator in hyperalgesia associated with inflammation [39,40]. Human studies correlate its synthesis with the cartilage degradation degree [41], and clinical trials demonstrate that reducing its expression results in a reduction of osteoarthritis pain [42]. Our study confirmed the increased cytokines’ expression and displayed that cashew nuts administration reduced pro-inflammatory cytokines’ serum levels and NGF expression as well. Destroyed joint homeostasis leads to catabolic processes, resulting in cartilage degeneration. From the histological point of view, cashew nuts’ oral administration reduced the radiographic and histological damage of knee joints. Cashew nuts also protected from loss of joint space, collagen, proteoglycans and articular cartilage erosion, induced by MIA injection [15,18,50]. Another reported characteristic of the osteoarthritis joints is the mast cells activation. They are an important source of cytokines, regulate vascular permeability and recruit other inflammatory cells, in particular leukocytes to osteoarthritis joints [51]. Oral administration of cashew nuts reduced mast cells’ infiltration in the knee joint induced by MIA injection.

## 5. Conclusions

Overall, our results showed that oral administration of cashew nuts reduced pain severity, restored the pro-oxidant/antioxidant balance and limited joint inflammation and tissue injury. They may be considered a valuable option to fight the osteoarthritis.

## References

[1] Grover A.K., Samson S.E. (2016). Benefits of antioxidant supplements for knee osteoarthritis: Rationale and reality. Nutr. J..

[2] Schaible H.G. (2012). Mechanisms of chronic pain in osteoarthritis. Curr. Rheumatol. Rep..

[3] Read S.J., Dray A. (2008). Osteoarthritic pain: A review of current, theoretical and emerging therapeutics. Expert Opin. Investig. Drugs.

[4] Forman H.J., Fukuto J.M., Torres M. (2004). Redox signaling: Thiol chemistry defines which reactive oxygen and nitrogen species can act as second messengers. Am. J. Physiol. Cell Physiol..

[5] Roehrs R., Guecheva T., Saffi J., Henriques J., ULBRA (2004). Redox sensitive targets in signaling cascades. Free Radicals and the Cellular Response to the Oxidative Stress.

[6] Altindag O., Erel O., Aksoy N., Selek S., Celik H., Karaoglanoglu M. (2007). Increased oxidative stress and its relation with collagen metabolism in knee osteoarthritis. Rheumatol. Int..

[7] Regan E.A., Bowler R.P., Crapo J.D. (2008). Joint fluid antioxidants are decreased in osteoarthritic joints compared to joints with macroscopically intact cartilage and subacute injury. Osteoarthr. Cartil..

[8] Hollman P.C., Cassidy A., Comte B., Heinonen M., Richelle M., Richling E., Serafini M., Scalbert A., Sies H., Vidry S. (2011). The biological relevance of direct antioxidant effects of polyphenols for cardiovascular health in humans is not established. J. Nutr..

[9] Shen C.L., Smith B.J., Lo D.F., Chyu M.C., Dunn D.M., Chen C.H., Kwun I.S. (2012). Dietary polyphenols and mechanisms of osteoarthritis. J. Nutr. Biochem..

[10] Baptista A., Goncalves R.V., Bressan J., Peluzio M. (2018). Antioxidant and Antimicrobial Activities of Crude Extracts and Fractions of Cashew (Anacardium occidentale L.), Cajui (Anacardium microcarpum), and Pequi (Caryocar brasiliense C.): A Systematic Review. Oxidative Med. Cell. Longev..

[11] da Silva R.A., Liberio S.A., do Amaral F.M., do Nascimento F.R.F., Torres L.M.B., Neto V.M., Guerra R.N.M. (2016). Antimicrobial and antioxidant activity of anacardium occidentale l. Flowers in comparison to bark and leaves extracts. J. Biosci. Med..

[12] Siracusa R., Fusco R., Peritore A.F., Cordaro M., D’Amico R., Genovese T., Gugliandolo E., Crupi R., Smeriglio A., Mandalari G. (2020). The Antioxidant and Anti-Inflammatory Properties of Anacardium occidentale L. Cashew Nuts in a Mouse Model of Colitis. Nutrients.

[13] Broinizi P.R., Andrade-Wartha E.R., Silva A.M., Torres R.P., Azeredo H.M., Alves R.E., Mancini-Filho J. (2008). Propriedades antioxidantes em subproduto do pedúnculo de caju (Anacardium occidentale L.): Efeito sobre a lipoperoxidação e o perfil de ácidos graxos poliinsaturados em ratos. Rev. Bras. de Ciências Farm..

[14] Morais T.C., Pinto N.B., Carvalho K.M., Rios J.B., Ricardo N.M., Trevisan M.T., Rao V.S., Santos F.A. (2010). Protective effect of anacardic acids from cashew (Anacardium occidentale) on ethanol-induced gastric damage in mice. Chem. Biol. Interact..

[15] Takahashi I., Matsuzaki T., Kuroki H., Hoso M. (2018). Induction of osteoarthritis by injecting monosodium iodoacetate into the patellofemoral joint of an experimental rat model. PLoS ONE.

[16] Lampropoulou-Adamidou K., Lelovas P., Karadimas E.V., Liakou C., Triantafillopoulos I.K., Dontas I., Papaioannou N.A. (2014). Useful animal models for the research of osteoarthritis. Eur. J. Orthop. Surg. Traumatol..

[17] Siracusa R., Impellizzeri D., Cordaro M., Peritore A.F., Gugliandolo E., D’Amico R., Fusco R., Crupi R., Rizzarelli E., Cuzzocrea S. (2020). The Protective Effect of New Carnosine-Hyaluronic Acid Conjugate on the Inflammation and Cartilage Degradation in the Experimental Model of Osteoarthritis. Appl. Sci..

[18] Di Paola R., Fusco R., Impellizzeri D., Cordaro M., Britti D., Morittu V.M., Evangelista M., Cuzzocrea S. (2016). Adelmidrol, in combination with hyaluronic acid, displays increased anti-inflammatory and analgesic effects against monosodium iodoacetate-induced osteoarthritis in rats. Arthritis Res. Ther..

[19] Britti D., Crupi R., Impellizzeri D., Gugliandolo E., Fusco R., Schievano C., Morittu V.M., Evangelista M., Di Paola R., Cuzzocrea S. (2017). A novel composite formulation of palmitoylethanolamide and quercetin decreases inflammation and relieves pain in inflammatory and osteoarthritic pain models. BMC Vet. Res..

[20] El-senosi Y.A. (2017). Biochemical studies on the effect of curcumin in experimentally induced osteoarthritis in rats. Benha Vet. Med. J..

[21] Cordaro M., Impellizzeri D., Siracusa R., Gugliandolo E., Fusco R., Inferrera A., Esposito E., Di Paola R., Cuzzocrea S. (2017). Effects of a co-micronized composite containing palmitoylethanolamide and polydatin in an experimental model of benign prostatic hyperplasia. Toxicol. Appl. Pharmacol..

[22] Ellman G.L. (1959). Tissue sulfhydryl groups. Arch. Biochem. Biophys..

[23] Uchiyama M., Mihara M. (1978). Determination of malonaldehyde precursor in tissues by thiobarbituric acid test. Anal. Biochem..

[24] Lawrence R.A., Burk R.F. (1976). Glutathione peroxidase activity in selenium-deficient rat liver. Biochem. Biophys. Res. Commun..

[25] Aebi H. (1980). Enzymes 1: Oxidoreductases, transferases. Methods Enzym. Anal..

[26] Gugliandolo E., D’Amico R., Cordaro M., Fusco R., Siracusa R., Crupi R., Impellizzeri D., Cuzzocrea S., Di Paola R. (2018). Effect of PEA-OXA on neuropathic pain and functional recovery after sciatic nerve crush. J. Neuroinflammation.

[27] Sarikcioglu L., Demirel B.M., Utuk A. (2009). Walking track analysis: An assessment method for functional recovery after sciatic nerve injury in the rat. Folia Morphol. (Warsz).

[28] Ahmed A.S., Li J., Erlandsson-Harris H., Stark A., Bakalkin G., Ahmed M. (2012). Suppression of pain and joint destruction by inhibition of the proteasome system in experimental osteoarthritis. Pain.

[29] D’amico R., Fusco R., Gugliandolo E., Cordaro M., Siracusa R., Impellizzeri D., Peritore A.F., Crupi R., Cuzzocrea S., Di Paola R. (2019). Effects of a new compound containing Palmitoylethanolamide and Baicalein in myocardial ischaemia/reperfusion injury in vivo. Phytomedicine.

[30] Cordaro M., Siracusa R., Impellizzeri D., D’Amico R., Peritore A.F., Crupi R., Gugliandolo E., Fusco R., Di Paola R., Schievano C. (2019). Safety and efficacy of a new micronized formulation of the ALIAmide palmitoylglucosamine in preclinical models of inflammation and osteoarthritis pain. Arthritis Res. Ther..

[31] Li G., Yin J., Gao J., Cheng T.S., Pavlos N.J., Zhang C., Zheng M.H. (2013). Subchondral bone in osteoarthritis: Insight into risk factors and microstructural changes. Arthritis Res. Ther..

[32] Billesberger L.M., Fisher K.M., Qadri Y.J., Boortz-Marx R.L. (2020). Procedural Treatments for Knee Osteoarthritis: A Review of Current Injectable Therapies. Pain Res. Manag..

[33] Philpott H.T., O’Brien M., McDougall J.J. (2017). Attenuation of early phase inflammation by cannabidiol prevents pain and nerve damage in rat osteoarthritis. Pain.

[34] Kesiktas F.N., Dernek B., Sen E.I., Albayrak H.N., Aydin T., Yildiz M. (2020). Comparison of the short-term results of single-dose intra-articular peptide with hyaluronic acid and platelet-rich plasma injections in knee osteoarthritis: A randomized study. Clin. Rheumatol..

[35] Radnovich R., Scott D., Patel A.T., Olson R., Dasa V., Segal N., Lane N.E., Shrock K., Naranjo J., Darr K. (2017). Cryoneurolysis to treat the pain and symptoms of knee osteoarthritis: A multicenter, randomized, double-blind, sham-controlled trial. Osteoarthr. Cartil..

[36] Ilfeld B.M., Ball S.T., Gabriel R.A., Sztain J.F., Monahan A.M., Abramson W.B., Khatibi B., Said E.T., Parekh J., Grant S.A. (2019). A feasibility study of percutaneous peripheral nerve stimulation for the treatment of postoperative pain following total knee arthroplasty. Neuromodulation.

[37] Guermazi A., Roemer F.W., Hayashi D., Crema M.D., Niu J., Zhang Y., Marra M.D., Katur A., Lynch J.A., El-Khoury G.Y. (2011). Assessment of synovitis with contrast-enhanced MRI using a whole-joint semiquantitative scoring system in people with, or at high risk of, knee osteoarthritis: The MOST study. Ann. Rheum. Dis..

[38] Hill C.L., Hunter D.J., Niu J., Clancy M., Guermazi A., Genant H., Gale D., Grainger A., Conaghan P., Felson D.T. (2007). Synovitis detected on magnetic resonance imaging and its relation to pain and cartilage loss in knee osteoarthritis. Ann. Rheum. Dis..

[39] McDougall J.J., Andruski B., Schuelert N., Hallgrimsson B., Matyas J.R. (2009). Unravelling the relationship between age, nociception and joint destruction in naturally occurring osteoarthritis of Dunkin Hartley guinea pigs. Pain.

[40] Schuelert N., McDougall J.J. (2009). Grading of monosodium iodoacetate-induced osteoarthritis reveals a concentration-dependent sensitization of nociceptors in the knee joint of the rat. Neurosci. Lett..

[41] Schomberg D., Ahmed M., Miranpuri G., Olson J., Resnick D.K. (2012). Neuropathic pain: Role of inflammation, immune response, and ion channel activity in central injury mechanisms. Ann. Neurosci..

[42] Ahmed S., Magan T., Vargas M., Harrison A., Sofat N. (2014). Use of the painDETECT tool in rheumatoid arthritis suggests neuropathic and sensitization components in pain reporting. J. Pain Res..

[43] Li D., Xie G., Wang W. (2012). Reactive oxygen species: The 2-edged sword of osteoarthritis. Am. J. Med. Sci..

[44] Henrotin Y., Kurz B., Aigner T. (2005). Oxygen and reactive oxygen species in cartilage degradation: Friends or foes?. Osteoarthr. Cartil..

[45] Ostalowska A., Birkner E., Wiecha M., Kasperczyk S., Kasperczyk A., Kapolka D., Zon-Giebel A. (2006). Lipid peroxidation and antioxidant enzymes in synovial fluid of patients with primary and secondary osteoarthritis of the knee joint. Osteoarthr. Cartil..

[46] Erturk C., Altay M.A., Selek S., Kocyigit A. (2012). Paraoxonase-1 activity and oxidative status in patients with knee osteoarthritis and their relationship with radiological and clinical parameters. Scand. J. Clin. Lab. Investig..

[47] Hassan M.Q., Hadi R.A., Al-Rawi Z.S., Padron V.A., Stohs S.J. (2001). The glutathione defense system in the pathogenesis of rheumatoid arthritis. J. Appl. Toxicol..

[48] Rubyk B.I., Fil’chagin N.M., Sabadyshin R.A. (1988). Change in lipid peroxidation in patients with primary osteoarthrosis deformans. Ter. Arkhiv.

[49] Zahan O.M., Serban O., Gherman C., Fodor D. (2020). The evaluation of oxidative stress in osteoarthritis. Med. Pharm. Rep..

[50] Liu P., Okun A., Ren J., Guo R.C., Ossipov M.H., Xie J., King T., Porreca F. (2011). Ongoing pain in the MIA model of osteoarthritis. Neurosci. Lett..

[51] Dean G., Hoyland J.A., Denton J., Donn R.P., Freemont A.J. (1993). Mast cells in the synovium and synovial fluid in osteoarthritis. Br. J. Rheumatol..

